# A novel joint analysis framework improves identification of differentially expressed genes in cross disease transcriptomic analysis

**DOI:** 10.1186/s13040-018-0163-y

**Published:** 2018-02-20

**Authors:** Wenyi Qin, Hui Lu

**Affiliations:** 1 Department of Bioengineering, University of Illinois at Chicago, 851 S. Morgan, Rm 218, Chicago, IL 60607 USA; 2 SJTU-Yale Joint Center for Biostatistics, Department of Bioinformatics and Biostatistics, Shanghai Jiaotong University, Shanghai, China; 3 Shanghai Engineering Research Center for Big Data in Pediatric Precision Medicine, Shanghai, China

**Keywords:** Public data integration, Cross disease transcriptome, Gene expression, Differentially expressed

## Abstract

**Motivation:**

Detecting differentially expressed (DE) genes between disease and normal control group is one of the most common analyses in genome-wide transcriptomic data. Since most studies don’t have a lot of samples, researchers have used meta-analysis to group different datasets for the same disease. Even then, in many cases the statistical power is still not enough. Taking into account the fact that many diseases share the same disease genes, it is desirable to design a statistical framework that can identify diseases’ common and specific DE genes simultaneously to improve the identification power.

**Results:**

We developed a novel empirical Bayes based mixture model to identify DE genes in specific study by leveraging the shared information across multiple different disease expression data sets. The effectiveness of joint analysis was demonstrated through comprehensive simulation studies and two real data applications. The simulation results showed that our method consistently outperformed single data set analysis and two other meta-analysis methods in identification power. In real data analysis, overall our method demonstrated better identification power in detecting DE genes and prioritized more disease related genes and disease related pathways than single data set analysis. Over 150% more disease related genes are identified by our method in application to Huntington’s disease. We expect that our method would provide researchers a new way of utilizing available data sets from different diseases when sample size of the focused disease is limited.

**Electronic supplementary material:**

The online version of this article (10.1186/s13040-018-0163-y) contains supplementary material, which is available to authorized users.

## Introduction

High-throughput technology like microarray and next-generation sequencing (NGS) allows researchers measure thousands of gene or microRNA expression in one sample simultaneously. Detecting differentially expressed (DE) genes between disease and normal control group is one of the most common analyses in genome-wide transcriptomic data. Differentially expressed genes are potential disease-related genes and could be used for generating biological hypothesis of disease mechanism, developing potential clinical diagnosis tools and investigating potential drug targets. This approach has been successfully applied in many complex diseases like cancers [10, 13] and diabetes [5, 33].

With the cost of microarray and next generation sequencing technique decreasing and stabilization of the experiment protocol, there are now over 1,000,000+ samples deposited in public databases such as Gene Expression Ominus (GEO) [6]. With this huge amount of public data available, it is now possible for researchers to perform cross disease transcriptomics comparison analysis. For example, Borjabad [1] compared the transcriptomes of postmortem brain tissues among HIV-associated neurocognitive disorders, Alzheimer’s disease and multiple sclerosis and found a large number of overlapped DE genes, indicating the shared mechanism among these three diseases which might lead to a common therapeutic approach. Swindell [27] identified common and specific gene signature in psoriasis by comparing the DE genes in psoriasis transcriptome with other DE genes of similar skin diseases. The cross disease transcriptomic analysis has provided researchers with new opportunities of understanding of mechanisms of complex disease and discovery of new biomarkers.

Numerous cross-disease analyses have shown that similar diseases might share similar disease related genes. However, in these cross-disease comparison studies, they took a simple “disease-by-disease” approach: each disease was analyzed with traditional DE detection method like two-sample t-test or *limma* [25] separately, then the overlap of DE genes between diseases was examined. This approach falls short in its ability to jointly analyze data on all diseases to improve the identification power while simultaneously considering for difference among DE genes present in each disease. Because of the incomplete power, this simple approach might lead to difficulties in interpreting the result of whether a gene is commonly shared by all disease or specific to one disease. On the other hand, joint analysis methods developed in other fields of omics data analysis and have been proven a useful method to increase the identification power by borrowing information from other similar diseases [2, 3, 16, 30].

Meta-analysis approach is a popular data integrating statistical methods used to analyze multiple public datasets of same biological conditions [20]. They improve the identification power by detecting the weak yet consistent signals through all studies of the same purpose. However, they are not suitable for cross-disease transcriptomic analysis because they assume that a gene is either differentially expressed in all studies or non-differential in all studies [9, 21, 22] while ignoring the context-specific signals within each disease study.

Motivated by this, we propose an empirical Bayesian based mixture model which jointly analyzed multiple similar diseases to increase the identification power of common and disease-specific DE genes. The rest of paper is organized as follows. First, through a comprehensive simulation study, we compare our method with single data set analysis as well as two popular meta-analysis approaches with different underlying null hypothesis: minP, maxP [31, 32] and demonstrate that our method outperforms these methods in terms of identification power. Then we apply our method to two real cases by jointly analyzing six microarray studies of different cancers as well as Alzherimer’s disease (AD) and Huntington’s disease (HD) show that joint analysis identifies more DE genes than single data set analysis and these DE genes are enriched with disease-related genes and pathways.

## Methods

### Joint analysis framework formulation

Assume that there are N data sets. Each data set contains both disease and normal samples. Same G genes’ expressions are measured in each data set. In the proposed joint analysis framework, a differential test is first performed for each gene *g* (*g* = 1, …*G*) to obtain a differential test score within each data set *i* (*i* = 1, …, *N*). In this study, we choose to use two sample t-statistic: *t*_*gi*_ as the differential test score. We then transform *t*_*gi*_ into a Z-score: *Z*_*gi*_ according to McLachlan’s normal transformation [17] so that the Z-score distribution of non-DE genes will be approximately normal. These Z-scores will serve as the basis of inference of DE in the joint analysis framework. We assume a two-component mixture model for all genes’ Z scores within each data set *i* where each gene’s hidden DE status variable *D*_*i*_ is either DE (*D*_*i*_ =1) or non-DE (*D*_*i*_ =0). Then we assume two different conditional density distributions of Z-scores depending on a gene’s hidden status *D*_*i*_ in data set *i*: *f*(*Z*| *D*_*i*_) where *D*_*i*_ =1 or 0. By doing so, we model the study-specific variation of Z-scores observed within each data set *i*.

Given observed expression difference $\overrightarrow{Z_g}=\left\{{Z}_{g1},{Z}_{g2}\dots, {Z}_{gN}\right\}$ between normal and disease groups across *N* disease datasets, we want to compute the posterior probability to infer the DE status of gene *g* in disease data set *i* which could be written as:

1 $$ \Pr \left({D}_i=1|{Z}_{g1},{Z}_{g2}\dots, {Z}_{gN}\right) $$

According to Bayes’ Theorem, we could expand (1) into:

2 $$ \Pr \left({D}_i=1|{Z}_{g1},{Z}_{g2}\dots, {Z}_{gN}\right)=\frac{\sum_{D_i=1}f\left({Z}_1,{Z}_2\dots, {Z}_N|{D}_1,{D}_2,\dots {D}_i=1,\dots {D}_N\right)\Pr \left({D}_1,{D}_2,\dots {D}_i=1,\dots {D}_N\right)}{f\left({Z}_1,{Z}_2\dots, {Z}_N\right)} $$

We further assume independence of conditional joint Z score distribution across data sets if the hidden status variable *D*_*i*_ is determined, written as:

$$ f\left({Z}_{g1},\dots, {Z}_{gN}|{D}_1,\dots {D}_i=1,\dots {D}_N\right)=f\left({Z}_{g1}|{D}_1\right)f\left({Z}_{g2}|{D}_2\right)\dots f\left({Z}_{gi}|{D}_i=1\right)\dots f\left({Z}_{gN}|{D}_N\right) $$

*f*(*Z*| *D*_*i*_) distribution is different from data set to data set, so it needs to be estimated separately for each data set. Here we apply the method of local false discovery rate (local FDR) developed by Efron [7] to estimate this conditional distribution. We refer interested readers to Efron’s paper for the details of the method. Here we just briefly describe the estimation procedure. The local FDR is written as:

3 $$ \mathrm{localFDR}\left({Z}_{gi}\right)=\Pr \left({D}_i=0|{Z}_{gi}\right)=\frac{f\left({Z}_{gi}|{D}_i=0\right)\Pr \left({D}_i=0\right)}{f\left({Z}_{gi}\right)} $$

where *f*(*Z*_*gi*_) = *f*(*Z*_*gi*_| *D*_*i*_ = 0) Pr(*D*_*i*_ = 0) + *f*(*Z*_*gi*_| *D*_*i*_ = 1) Pr(*D*_*i*_ = 1) and Pr(*D*_*i*_ = 1) =1 − Pr(*D*_*i*_ = 0).

In the localFDR approach, the marginal density *f*(*Z*_*gi*_) is estimated through fitting z-scores of all genes to a cubic spline. The conditional density *f*(*Z*_*gi*_| *D*_*i*_ = 0) is assumed to be a normal distribution. Its mean and variance as well as the quantity Pr(*D*_*i*_ = 0) are estimated through fitting the Z-scores in the central peak (around 0) by maximum likelihood estimation approach. This is a reasonable assumption because most Z-scores around the 0 should come from the non-DE distribution. Then through Eq. (3), we could also obtain the estimate of *f*(*Z*_*gi*_| *D*_*i*_ = 1). All the estimation procedures described above are done through the *locfdr* package in R [8]. In this study, we also use Pr(*D*_*i*_ = 1| *Z*_*gi*_) = 1− localFDR(*Z*_*gi*_) computed by local FDR method as the inference result of single data set analysis for method comparison purpose.

Finally, we need to estimate the only unknown parameter in Eq. (2), the prior probability of a gene’s DE status in different diseases: Pr(*D*_1_, *D*_2_, …*D*_*i*_, …*D*_*N*_). The shared information between similar diseases is also modeled by this quantity. For example, we would expect that if one gene is DE in one disease, it is highly likely to be DE in another similar disease. In mathematics, we write this relation as Pr(*D*_1_ = 1, *D*_2_ = 1) = Pr(*D*_2_ = 1| *D*_1_ = 1) Pr(*D*_1_ = 1) and Pr(*D*_1_ = 1, *D*_2_ = 1) ≠ Pr(*D*_1_ = 1) Pr(*D*_2_ = 1). This prior probability could be estimated by using Expectation Maximization (EM) algorithm [4] to maximize the marginal log likelihood of all genes’ expression Z-scores in all data sets. EM algorithm steps could be summarized as follows:

1. Initialize the prior probability: ${\Pr}^{(0)}\left({D}_1,{D}_2,\dots {D}_i,\dots {D}_N\right)=\frac{1}{2^N}$.
2. At iteration *s*, compute the joint posterior probability of gene *g* given $\overrightarrow{Z_g}$:

$$ {\Pr}^{(s)}\left({D}_1,\dots, {D}_N\ |{Z}_{g1},\dots, {Z}_{gN}\right)=\frac{f\left({Z}_{g1},\dots, {Z}_{gN}|{D}_1,\dots, {D}_N\right){\Pr}^{(s)}\left({D}_1,\dots, {D}_N\right)}{f\left({Z}_{g1},\dots, {Z}_{gN}\right)} $$

$$ where\ f\left({Z}_{g1},\dots, {Z}_{gN}|{D}_1,\dots, {D}_N\right)=\prod \limits_{i=1}^Nf\left({Z}_{gi}|{D}_i\right) $$

$$ f\left({Z}_{g1},\dots, {Z}_{gN}\right)=\sum \limits_{\Pr^{(s)}\left({D}_1,\dots, {D}_N\right)}f\left({Z}_{g1},\dots, {Z}_{gN}|{D}_1,\dots, {D}_N\right){\Pr}^{(s)}\left({D}_1,\dots, {D}_N\right) $$

(3) Estimate the new prior probability at iteration s + 1 by averaging the joint probability calculated in step (2) over all genes: ${\Pr}^{\left(s+1\right)}\left({D}_1,\dots, {D}_N\right)=\frac{1}{G}\sum \limits_{g=1}^G{\Pr}^{(s)}\left({D}_1,\dots, {D}_N\ |{Z}_{g1},\dots, {Z}_{gN}\right)$
(4) Repeat step (2) and (3) until convergence.

The proposed joint analysis framework is implemented under R statistical programming language.

### Simulation studies

In real world, researchers often have limited samples for a specific disease and do not have other public data sets of the same disease while public data set of other similar diseases exists. We design a simulation study which mimics the real situation to test and compare the performance of our method with others. Our simulation study models this situation by generating different number of studies with similar but slightly different DE gene configuration in each disease.

To be more specific, the simulation is set to have *N* studies with 15 disease and 15 control samples within each study. Each study here could be considered as a similar disease. There is a total of 10,000 genes expression value measured in each sample. We first need to determine the hidden DE status variable value for each gene in each study. We define *shared percentage* between study *i* and study *j* as the conditional probability of being DE in study *j* if the hidden DE status variable in study *i* is true, i.e. Pr(D_*j*_ = 1 ∣ D_*i*_ =1). We further define “similarity” as the average shared percentage of DE genes between two studies, i.e. $\frac{1}{2}\left(\Pr \left({\mathrm{D}}_j=1|{\mathrm{D}}_i=1\right)+\Pr \left({\mathrm{D}}_i=1|{\mathrm{D}}_j=1\right)\right)=\frac{1}{2}\left(\frac{\Pr \left({D}_i=1,{D}_j=1\right)}{\Pr \left({\mathrm{D}}_i=1\right)}+\frac{\Pr \left({D}_i=1,{D}_j=1\right)}{\Pr \left({\mathrm{D}}_j=1\right)}\right)$. We also assume there is around 10% of DE genes in each study i.e. Pr(D_*i*_ =1) = 0.1. We finally define two diseases are “similar” if the similarity value between two diseases is higher than the expected similarity (i.e. Pr(*D*_*i*_ = 1, *D*_*j*_ = 1) = Pr(D_*i*_ = 1) Pr(D_*j*_ = 1)). Therefore, once the DE status variable value of a gene in the reference study is known, we could generate DE status variables of this gene for all other studies. In this simulation, we assume study 1 is the reference study, the hidden DE status configuration of other studies is then generated for each gene based on the DE status variable in study. After the hidden DE status variable is determined, we generate the normally distributed expression value based on the DE status of each gene. The variance σ^2^_*gd*_ of each gene *g* is assumed to be the same in each study *d* and is sampled from an inverse chi-square distribution with degrees of freedom 4 and scale parameter 0.02. We then generate gene ’s expression for every sample from N(0, σ^2^_*gd*_). If D_*i*_ =1, we sample a μ_*gd*_ from N(0, *w*_*gd*_ ∗ σ^2^_*gd*_) where *w*_*gd*_ = 4 here and add it to the expression value of disease samples. By using this simulation setup, we mimic the real case when the sample size of target disease is small but studies of similar diseases exist in public database.

We also design another simulation study by fixing the hidden DE status of each gene before generating the expression value. In this simulation study, we assume that there are *N* = 2 data sets, with same number of genes and samples setup described above. In the first data set, the first 1000 genes are assumed to be DE. In the second data set, we assume that first *X* genes are DE and for the rest of *1000-X* genes, we ensure that they will not overlap with any DE genes in data set 1. Once the DE status of all genes are set in two data sets, gene expression values are generated with the same procedure described above. By setting so, the true prior probability is a fixed value. For example, if X = 600, then the prior probability will be Pr(D_1_ = 1, D_2_ =1) =0.06, Pr(D_1_ = 0, D_2_ =1) =0.04, Pr(D_1_ = 1, D_2_ =0) =0.04 and Pr(D_1_ = 0, D_2_ =0) = 0.86 respectively. By using this simulation setup, we are able to compare the estimated prior probability generated from joint analysis with the true value.

### Meta-analysis methods

Two popular meta-analysis approaches are compared with our joint analysis method: minP and maxP [28]. These two methods represent two different underlying hypothesis used in meta-analysis methods: the first hypothesis tests if one gene is DE in at least one or more data sets or not; the second hypothesis detects if one gene is DE in all studies or not [28]. Briefly speaking, The maxP method takes maximum of *p*-value from each study as test statistics: *S*_*g*_^*maxP*^ = *argmax*(*p*_*gk*_). *S*_*g*_^*maxP*^ follows a beta distribution with degrees of freedom α = *N* and β = 1 under null hypothesis. The maxP method targets the DE genes with small *p*-values in all studies. The minP method takes the minimum p-value among the K studies as the test statistic: *S*_*g*_^*minP*^ = *argmin*(*p*_*gk*_). It follows a beta distribution with degrees of freedom α = 1 and β = N under the null hypothesis. This method detects a DE gene whenever a small p-value exists in any one of all studies. All meta-analysis methods used in this paper are implemented through using *metaRawdata()* function in *metaDE* R package [31, 32]. Two sample t-test is used as summary statistic for each individual study and parametric assumption is used to obtain the p-value of the statistic.

### Real data application

#### Cancer data sets

Six normalized microarray expression data sets representing different types of cancers are downloaded from GEO [6]. Each data set contains at least 25 control samples of normal tissues. The GEO accession number and details of each data set are summarized in Table 1. The joint analysis and single data set analysis are applied to the real data set and evaluated based on the number of identified genes with a pre-defined cutoff and the number of cancer related genes by using a 743 cancer-related gene lists compiled by Nagaraj [18]. The probe list in each microarray platform is first converted to gene symbol and same genes are extracted from each platform. Twelve thousand four hundred sixty-six genes are found common to all microarray platforms and will be used in this study.

**Table 1:** Summaries of six different cancer data sets used in this study

Dataset ID	Disease Name	Microarray Platforms	# of disease samples	# of control samples	Reference
GSE13507	Bladder cancer	GPL6102	165	68	[13]
GSE41258	Colorectal cancer	GPL96	181	58	[24]
GSE19188	NSCLC	GPL570	91	65	[10]
GSE9476	AML	GPL96	26	38	[26]
GSE32863	Lung adenocarcinoma	GPL6884	58	58	[23]
GSE1542	Pancreatic Cancer	GPL96	24	25	[12]

Abbreviations: *NSCLC* Non-Small Cell Lung Carcinoma, *AML* Acute Myelocytic Leukemia

#### Alzheimer’s disease and Huntington’s disease data sets

Narayanan et al. conducted a co-expression network analysis between Alzheimer’s disease (AD) and Huntington’s disease (HD) using the prefrontal cortex region of postmortem brain samples consisting of 310 AD patients, 157 HD patients and 157 controls [19]. The microarray expression data is deposited at GEO (GEO Accession no: GSE33000) and downloaded. A linear model is then fit to each gene with gender, age and hidden batch variables estimated with *sva* R package [15] as covariates to correct for confounding factors. The t-statistic of disease effect of each gene is then extracted. Single data set analysis and joint analysis are then applied to the t statistics and DE results are obtained for each disease. A total of 39,280 probes (some probes will represent the same gene) are measured and will be used in this study.

## Results

### Formulation of proposed joint analysis framework

**Fig. 1:**
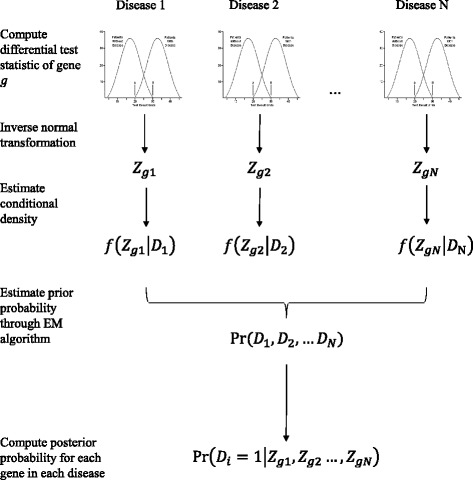
Workflow of proposed joint analysis framework

The workflow of the joint analysis framework is shown in Fig. 1. The framework could be broken down into the following steps: The first step is to compute a differential test statistic for each gene *g* in each data set *i*, then the differential test statistics is transformed into a Z-score: *Z*_*gi*_. Then within each data set, estimate *f*(*Z*| *D*_*i*_) distribution with localFDR approach. After the conditional density value is obtained for each *Z*_*gi*_, the prior probability Pr(*D*_1_, *D*_2_, …*D*_*i*_, …*D*_*N*_) is estimated through EM algorithm. Finally compute posterior probability defined in Eq. (2) in Methods section for each gene in each data set and genes are ranked based on this quantity. A gene would be called DE if the posterior probability is higher than some pre-defined threshold.

### Simulation studies

#### Comparison between joint analysis and single data set analysis

We begin by comparing the identification power between single data set analysis and joint analysis using simulation studies. Different simulated disease data sets are generated by varying the number of data sets and shared percentage among diseases as described in “Methods” section. The number of data sets is set for *N* = 1, 2, 4, 6 and the shared percentage between study 1 and other studies is set to Pr(D_*j*_ = 1 ∣ D_1_ =1) = 0,0.1, 0.6, 0.7, 0.8, 0.9, 1. Every parameter combination is repeated for 100 times. In each run, we set a specified posterior probability cutoff in data set 1 which is considered as the disease data set of interest and report the average sensitivity as well as average false discovery rate (FDR) in study 1 as the result. The cutoff is set to 0.95.

**Fig. 2:**
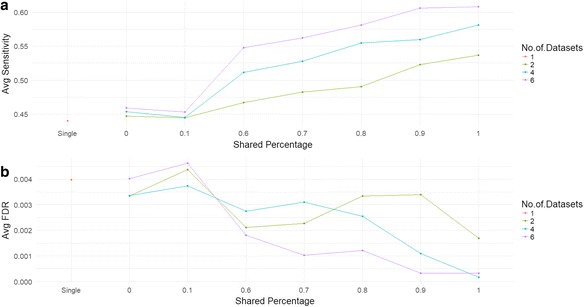
Average sensitivity and False Discovery Rate (FDR) comparison between single data set analysis and joint analysis under different simulation parameter setup. The results are summarized from 100 runs. **a** Average sensitivity comparison. **b** Average FDR comparison

Figure 2 shows the results of average sensitivity and average FDR comparison between joint analysis and single data set analysis. By setting N = 1, we are comparing the identification power of joint analysis with single data set analysis and improved identification power is expected to be observed when two diseases shared a larger proportion of DE genes; by setting shared percentage to 0 and 0.1, this could be regarded as integrations of two diseases with no overlapping DE genes and two random diseases and we would expect that no power improvement and the result of joint analysis should be similar to single data set analysis. From Fig. 2a, we observe that when the value of shared percentage increases, which suggests that the similarity between diseases increases, the sensitivity increases, more true DE genes could be prioritized than separately analyzing one disease data set. Also, if the number of similar disease data sets increases, the joint analysis could borrow more shared information from other disease data sets and thus have a higher average sensitivity than those with less number of data sets. We tested different posterior probability cutoffs (0.9, 0.8 and 0.5) and the results are very similar to what are observed here (Additional file 1: Table S1). We further examined the average FDR in single data set analysis and joint analysis respectively. The results shown in Fig. 2b indicate that joint analysis with increased shared percentage and increased number of data sets do not come at the cost of increasing the number of false positives. The results shown here demonstrated the improved identification power of joint analysis over single data set analysis by borrowing shared information from other similar diseases and the identification power would increase when more similar disease data sets are available while the false discovery rate is under control.

#### Comparison with other meta-analysis approaches

**Fig. 3:**
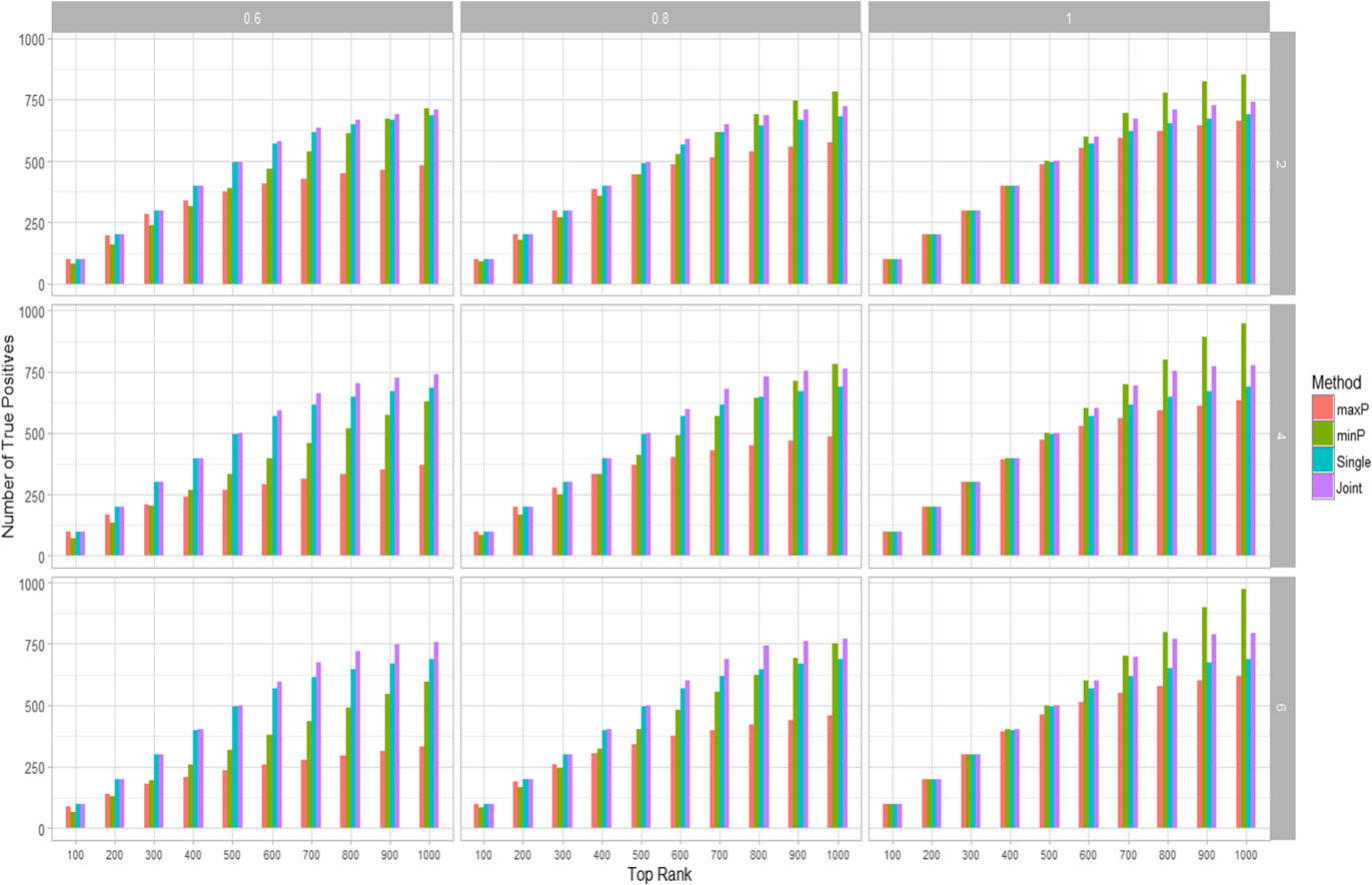
Number of true genes against top ranked genes evaluated by different methods under different simulation parameter setup: shared percentage = 0.6, 0.8, 1; number of data sets = 2, 4, 6

We then compared the proposed joint analysis framework with other two popular meta-analysis approaches: minP and maxP and use single data set analysis as a baseline of comparison. We evaluated the performance of different methods by plotting the top ranked genes against the average number of true DE genes identified in study 1 out of 100 runs with varying values of shared percentage and number of data sets. The number of data sets is set for *N* = 2, 4, 6 and the shared percentage between study 1 and other studies is set to Pr (D_j_=1|D_1_ = 1) = 0.6, 0.8, 1. The results are shown in Fig. 3. When the shared percentage is set to 0.6, the joint analysis consistently outperforms all other methods by identifying more true DE genes in top 1000 genes except in the rank range of 900 to 1000 when *N* = 2 minP and joint analysis have similar performance. When the shared percentage value increases to 0.8, joint analysis outforms other methods in top ranking below 800, minP method performs better in the rank range of top 800–1000 genes. When the shared percentage is 1, which means all measurement are based on same disease, minP has better performance. Overall, when the data sets are based on similar but different diseases, especially when more diseases are included, our joint analysis outperformed other methods.

#### Evaluation of estimated prior probability

The estimated prior probability from joint analysis is evaluated because prior probability plays an important role in empirical Bayes framework. We first compared the estimated prior probability with true value in a two-dataset simulation study in which hidden DE status of genes were fixed. The details of the simulation was described in “Methods” section. *X* value was set to 600, 700, 800, 900 and 1000. Each parameter setup was repeated 10 times and the results were summarized in Table 2. By comparing the estimated value with true value, we observed that the joint analysis framework will underestimate the shared percentage of genes but had an increasing trend when the shared number of genes increases. Further, when the number of data sets increased to 4 and 6, we compared the similarity estimate obtained from joint analysis with true similarity value as defined in the “Methods” section between data set 1 and data set 2. The simulation setup was the same as that in the sensitivity and FDR comparison and the results of comparison were similar (Additional file 2: Table S2). The main reason for the observed conservative estimate of shared gene pairs might be that the local false discovery method implemented in the joint analysis framework tends to be conservative by classifying most genes at the boundary between the null distribution and alternative distribution to the null distribution so that the shared gene pairs at the boundary might be difficult to be correctly classified. This problem could be alleviated by employing parametric distribution setup for the joint analysis framework but the current non-parametric framework is more general and could be used in more situations. Nevertheless, the accurate estimation of increasing trend of shared gene pairs could help the joint analysis to put correct priors among diseases to infer DE status of a gene.

**Table 2:** Comparison of estimated prior probability with true ratio in the simulation study

DE status	X									
	600		700		800		900		1000	
	Estimate	Truth	Estimate	Truth	Estimate	Truth	Estimate	Truth	Estimate	Truth
(0,0)	0.8857 (0.006)^a^	0.86	0.8914 (0.006)	0.87	0.8982 (0.006)	0.88	0.9054 (0.005)	0.89	0.9068 (0.003)	0.9
(0,1)	0.041 (0.005)	0.04	0.0356 (0.005)	0.03	0.0284 (0.004)	0.02	0.0218 (0.003)	0.01	0.018 (0.003)	0
(1,0)	0.042 (0.008)	0.04	0.0365 (0.008)	0.03	0.0316 (0.006)	0.02	0.0258 (0.003)	0.01	0.02 (0.004)	0
(1,1)	0.031 (0.003)	0.06	0.0364 (0.002)	0.07	0.0417 (0.004)	0.08	0.0469 (0.005)	0.09	0.0546 (0.004)	0.1

^a^ The values in the parentheses represent the standard deviation summarized from 10 repeated runs

#### Influence of sample size of a similar disease

**Fig. 4:**
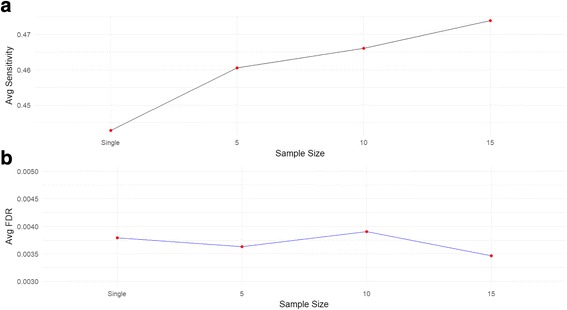
Influence of sample size of a similar disease to be borrowed from. The results are averaged from 100 runs for each sample size parameter setup. **a** Average sensitivity comparison. **b** Average FDR comparison

Finally, the influence of sample size of a similar disease to be borrowed from is evaluated. To achieve this purpose, we first fix the target data set with 15 disease and 15 control samples. Then, we generate second similar (60% similarity) disease data sets with different sample sizes, each of which contains 5, 10 and 15 disease and control samples respectively. The mean and variance for each gene in each data set is fixed in this simulation. This simulation procedure is then repeated 100 times for each sample size parameter. After that, we apply both single and joint analysis on the simulated data sets and record the average sensitivity and FDR at specified cutoff = 0.95 for each sample size parameter. The result is shown in Fig. 4. As expected, the average sensitivity increases as the sample size increases. The average FDR is well controlled and only shows very small fluctuation due to sampling error in generating expression values for each gene. In conclusion, the simulation results demonstrate that the proposed joint analysis framework could borrow more information from a similar disease of a larger sample size.

### Real data application: six different cancers

**Fig. 5:**
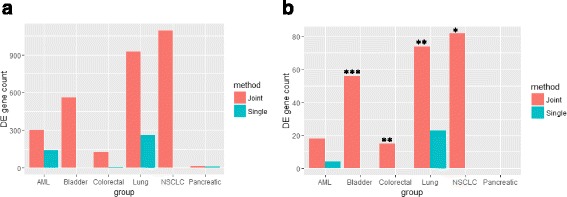
DE gene identification results comparison between joint analysis and single data set analysis on six cancer data sets. **a** The total number of identified DE genes in each cancer. **b** The number of cancer-related DE genes identified in each cancer. The enrichment level is evaluated by the *p*-value of hypergeometric test. ***: < 0.001; **: < 0.01; *: < 0.05

We considered cancer as a sample study because many genes were observed commonly dysregulated in different cancers suggesting certain shared mechanisms regardless of the source of tissue type [21, 29]. We applied the joint analysis on six public data sets of different cancers and compare the DE gene identification results with those obtained by single data set analysis using the same predefined posterior probability cutoff of 0.95. The results are summarized in Fig. 5. In Fig. 5a, we saw a significant identification power gain in NSCLC and lung adenocarcinoma. A moderate gain of power was observed in bladder cancer, colorectal cancer and AML. Little gain of power was observed in pancreatic cancer. The DE gene results obtained by single data set analysis and joint analysis in AML and lung adenocarcinoma data sets were then compared. We observed that all genes identified by the single data set analysis could also be identified by the joint analysis. The complete DE gene lists of the joint analysis could be viewed in Additional file 3: Table S3. We further examined the overlapped percentage of identified genes between single data set and joint analysis in our previous simulation study with *N* = 6 and increased shared percentage parameter setup with cutoff = 0.95. The simulation study suggested that the joint analysis could identify most of genes which are identified by single data set analysis (Additional file 4: Table S4). The comparison results of cancer data sets were thus consistent with those in simulation studies and demonstrated that our proposed joint analysis framework could identify most of genes that are also identified by single data set analysis with improved identification power.

To further validate the biological relatedness of identified DE genes, we also checked if the DE gene lists are enriched with cancer-related genes by comparing the DE gene lists with a 743 known cancer-related gene lists compiled by Nagaraj [18]. The results in Fig. 5b showed that the joint analysis identifies more cancer-related genes than single data set analysis and hypergeometric test shows that the newly identified DE genes are enriched with cancer-related genes in bladder, colorectal, lung and NSCLC data sets respectively while there is no enrichment seen in the results of single data set analysis using the same cutoff.

**Fig. 6:**
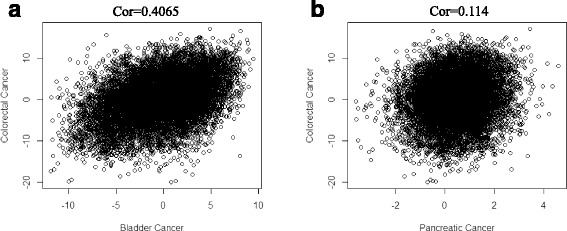
Scatterplot of Z-scores between two cancers. **a** Scatterplot of Z-score pairs between colorectal cancer and pancreatic cancer. **b** Scatterplot of Z-score pairs between colorectal cancer and bladder cancer

We then examined the correlations relationship of same genes between cancers to understand how information is shared across cancers. We plotted the pair of Z-scores obtained from colorectal and pancreatic cancer data sets as well as colorectal and bladder cancer data sets as a reference (Figure 6). Pearson’s correlation coefficient is also computed for each pair of cancers. A weak correlation is observed in Z-score pairs between pancreatic cancer and colorectal cancer while there is a strong correlation between bladder cancer and colorectal cancer. The result might explain part of the reason why there is little gain of power in pancreatic cancer data set through joint analysis.

Finally, we computed the pair-wise similarity between each cancer with estimated prior probability and the result is shown in Table 3. As expected, the pancreatic cancer shared the fewest DE genes with other cancers so that few information could be borrowed. The lung adenocarcinoma and NSCLC shared largest percentage of DE genes as their origins are the same. The bladder cancer, colorectal cancer and lung adenocarcinoma shared a large percentage of DE genes mainly because these cancers all belong to the category of adenocarcinoma and might share a common underlying dysregulated pathway. AML showed moderate sharing percentage with other cancers probably because the origin of the cancer is different from others. Thus, the joint analysis framework could provide a reasonable inference on DE gene similarity between cancers.

**Table 3:** Pair-wise similarity estimated among cancers

	Bladder	Colorectal	NSCLC	AML	Lung	Pancreatic
Bladder	1	0.521	0.525	0.219	0.435	0.132
Colorectal	0.521	1	0.511	0.168	0.486	0.117
NSCLC	0.525	0.511	1	0.251	0.906	0.148
AML	0.219	0.168	0.251	1	0.197	0.085
Lung	0.435	0.486	0.906	0.197	1	0.124
Pancreatic	0.132	0.117	0.148	0.085	0.124	1

### Real data application: Alzheimer’s disease and Huntington’s disease

We take Alzheimer’s disease and Huntington’s disease as another sample study because these two neurodegenerative diseases are found to share very similar pathology and phenotypes [19]. We applied a linear model for each gene as described in Methods section to correct for the influence of covariate and hidden batch effect. The t-statistic of disease effect is then extracted and fed into both single data set and joint analysis frameworks. We obtained the ranked DE gene lists and compare them against genes along AD and HD pathways defined in KEGG data base respectively. The results are shown in Table 4. In the case of AD, the joint analysis approach showed a moderate borrowing of information from Huntington’s disease by consistently prioritizing more genes along AD pathways among top ranked DE genes. In HD, we observed a much larger gain of power. Among top 250, 500 and 1000 range, we obtained 160, 171 and 168% more HD-related genes in joint analysis framework than analyzing the data set alone. The improvement is mainly due to a high percentage of shared DE genes between AD and HD (around 9% of total genes) and the examination of prior probability estimate confirmed that there might be only a very small percentage of HD-specific DE genes (data not shown). We also checked the overlapping genes between single data analysis and joint analysis, there is a total of 17 genes commonly identified by both methods. For the 15 HD related genes exclusively identified by joint analysis, we examined their posterior probability value and ranks in both single data set analysis and joint analysis and the results are shown in Table 5. We observed that the statistical evidence and the ranks of these genes are significantly improved by joint analysis. The average posterior probability gain is 0.214 and the average rank improvement is 692.2. These results clearly demonstrate that our proposed joint analysis framework has improved identification power over single data set analysis and could also recover most of genes that are identified by single data set analysis. We further examined the KEGG pathway enrichment of top ranked genes in HD to examine the possible biological roles of these top ranked DE genes. Top 1000 genes obtained by single data analysis and joint analysis are submitted to DAVID [11] server to perform the pathway enrichment analysis respectively. The top 10 KEGG pathway enrichment results are ordered by their raw enrichment *p*-values. The number of DE genes identified along the pathway, the raw enrichment *p*-value of the pathway and Bonferroni’s corrected *p*-value are reported in Table 6. Table 6 showed that the enriched KEGG pathways obtained by single analysis and joint analysis have a large overlap. The joint analysis prioritized three similar neurodegenerative disease related pathways and their corresponding biological process: oxidative phosphorylation over single data set analysis by identifying more DE genes along those pathways shared by these diseases. Metabolic pathways are found to be differentially expressed in several neurodegenerative disorders such as in schizophrenia [20] and identified as more enriched in joint analysis. It is worth noting that synaptic vesicle cycle pathway, which is closely related to neurotransmitter release and neurodegenerative disorders [34], is exclusively identified by joint analysis.

**Table 4:** Number of genes in KEGG pathway of AD and HD among top ranked genes in each neurodegenerative disorder

	Alzheimer’s Disease		Huntington’s disease	
Top Rank	Single	Joint	Single	Joint
< 250	2	4	5	6
< 500	9	12	10	16
< 750	19	21	14	24
< 1000	29	30	19	32

**Table 5:** Posterior probability and rank comparison of 15 HD-related genes exclusively identified by joint analysis among top 1000 genes

Gene Symbol	Single P^a^	Joint P^b^	Single Rank	Joint Rank
ATP5B	0.722686833	0.938701726	1475	652
ATP5F1	0.712111394	0.933163998	1690	954
ATP5G1	0.724132275	0.939691574	1443	594
ATP5J	0.71465263	0.935545911	1639	827
CLTA	0.704691436	0.933779808	1833	918
COX4I1	0.732382138	0.938415838	1208	673
NDUFA7	0.718914525	0.938173615	1553	683
NDUFA9	0.738565931	0.942103957	1037	460
NDUFB5	0.731956544	0.940700886	1218	546
NDUFB6	0.709427659	0.932649277	1741	972
POLR2K	0.705645646	0.935145538	1806	850
SLC25A5	0.737126871	0.934489089	1089	884
UQCRC1	0.727082935	0.932648306	1373	973
UQCRH	0.723833736	0.934133476	1448	902
VDAC2	0.738319827	0.944168188	1045	327

^a^ Posterior probability of true DE status in single data set analysis

^b^ Posterior probability of true DE status in joint analysis

**Table 6:** Top 10 KEGG pathway enrichment results comparison between (A) single data set analysis and (B) joint analysis in Huntington’s disease

Term	Count	Enrichment Pvalue	Bonferroni corrected P
(A) Single			
hsa03050:Proteasome	12	5.14E-07	1.21E-04
hsa05010:Alzheimer’s disease	20	1.95E-05	0.004579209
hsa05012:Parkinson’s disease	18	2.58E-05	0.006034046
hsa05016:Huntington’s disease	19	3.66E-04	0.08242395
hsa05033:Nicotine addiction	7	0.003777846	0.589128604
hsa00190:Oxidative phosphorylation	13	0.004721495	0.671158323
hsa04932:Non-alcoholic fatty liver disease (NAFLD)	14	0.00497103	0.689975889
hsa05169:Epstein-Barr virus infection	15	0.013743654	0.9613094
hsa04723:Retrograde endocannabinoid signaling	10	0.01471692	0.969321024
hsa04728:Dopaminergic synapse	11	0.02422846	0.996860832
(B) Joint			
hsa05012:Parkinson’s disease	29	3.18E-13	7.59E-11
hsa00190:Oxidative phosphorylation	27	2.90E-12	6.92E-10
hsa05016:Huntington’s disease	32	4.34E-12	1.04E-09
hsa05010:Alzheimer’s disease	29	2.33E-11	5.57E-09
hsa04932:Non-alcoholic fatty liver disease (NAFLD)	22	2.14E-07	5.12E-05
hsa03050:Proteasome	10	3.21E-05	0.007653523
hsa01100:Metabolic pathways	73	5.47E-05	0.012999463
hsa05169:Epstein-Barr virus infection	20	1.02E-04	0.024158392
hsa04721:Synaptic vesicle cycle	10	5.70E-04	0.127417187
hsa01200:Carbon metabolism	13	0.001156092	0.241540496

## Conclusion and discussion

In this paper, we present a novel statistical framework which aims at addressing a problem often met by biological researchers: when only a limited number of sample for a specific disease is available, the identification power could be improved by jointly analyzing multiple similar disease data sets because DE genes might be shared among similar diseases. By implementing a two-component mixture model, we demonstrate the framework could improve the identification power through comprehensive simulation studies and two real data applications. The joint analysis outperforms single data set analysis in both identification power and biological interpretation.

The prior probability is the most essential quantity in the proposed joint analysis framework and has a large impact on the performance of the method because similarity between diseases are directly determined by this quantity. This has been demonstrated through both simulation study and real data application. In simulation studies, we observed that when jointly analyzed with diseases with higher similarity, which was realized by adjusting prior probability value among diseases, the target data set gained more statistical power than less similar diseases. In real data application, more DE genes were identified among similar cancers than dissimilar ones where similarity among cancers were computed through estimated prior probability. In short, prior probabilities among different diseases could determine if the proposed joint analysis framework would be effective or not.

There would be several improvements for the proposed joint framework in the future. The first issue to be addressed is how to jointly analyze more disease data sets. As mentioned by one reviewer, the estimation of the prior probability in the proposed framework here is computationally intensive when the number of diseases to be jointly analyzed is large (~2^N^, where N is the total number of diseases). The estimation of prior probability would become infeasible when the number reaches 20 or more. Some potential solution to this problem has been proposed in a recent paper [14]. The basic idea is to assume special structures about the prior probability such that the number of prior probability to be estimated could be significantly reduced, thus incorporating more disease data sets becomes available. Another improvement would be to design a disease similarity test so that researchers could determine if two diseases are similar enough to be jointly analyzed. A similar idea has been proposed by Chung et al. [3] where a likelihood test was designed to evaluate if two diseases contain similar SNPs. Finally, next generation sequencing support is expected to be added to current framework such that microarray and sequencing data could be analyzed simultaneously.

## Additional files

Additional file 1: Table S1. Comparison of average sensitivity and FDR between single and joint analysis. (XLSX 16 kb)

Additional file 2: Table S2. Comparison of estimated similarity from joint analysis and true similarity. (XLSX 8 kb)

Additional file 3: Table S3. Complete DE gene lists identified by joint analysis. (XLSX 81 kb)

Additional file 4: Table S4. Overlapped genes comparison between single data set and joint analysis. (XLSX 8 kb)
